# Effect of donepezil on transcranial magnetic stimulation parameters in Alzheimer's disease

**DOI:** 10.1016/j.trci.2018.02.001

**Published:** 2018-03-02

**Authors:** Yun Tae Hwang, Lorenzo Rocchi, Paul Hammond, Chris JD. Hardy, Jason D. Warren, Basil H. Ridha, John Rothwell, Martin N. Rossor

**Affiliations:** a Institute of Neurology, University College London, London, United Kingdom; b Brain and Mind Centre, University of Sydney, Camperdown, New South Wales, Australia; c NIHR Queen Square Dementia Biomedical Research Unit, Institute of Neurology, University College London, London, United Kingdom; d Dementia Research Centre, Institute of Neurology, University College London, London, United Kingdom

**Keywords:** Alzheimer's disease, Response to treatment, Transcranial magnetic stimulation, Short-latency afferent inhibition, Acetylcholinesterase inhibitor, Donepezil

## Abstract

**Introduction:**

There is a need for a reliable, noninvasive biomarker for Alzheimer's disease (AD). We assessed whether short-latency afferent inhibition (SAI), a transcranial magnetic stimulation paradigm that assesses cholinergic circuits of the brain, could become such a biomarker.

**Methods:**

Nineteen patients with AD underwent four SAI testing sessions. The timing of their usual donepezil dose was altered to create different cholinergic states for each session. This was compared to the SAI results from 20 healthy subjects.

**Results:**

SAI was not able to distinguish the different cholinergic states assessed in our study. There appeared to be a diurnal variation in cholinergic function in the control group, which was not present in the AD cohort.

**Discussion:**

SAI does not appear to have a role in diagnosis and assessment of AD patients. The loss of diurnal variation, however, warrants further investigation as it may provide further biochemical insights about AD.

## Highlights

• Transcranial magnetic stimulation is unable to differentiate various cholinergic states in Alzheimer's disease patients on donepezil.
• Chronic donepezil therapy may induce neuroplastic changes in hippocampal neurons.
• A diurnal variation in cholinergic activity may be present in healthy people.
• Alzheimer's disease may lead to loss of this diurnal cholinergic activity variation.

## 1 Introduction

Alzheimer's disease (AD) is the most common neurodegenerative disorder affecting cognition, usually beginning with memory impairment and executive dysfunction [1]. Biochemically, it is characterized by a cholinergic deficit in the brain [2]. Increasing acetylcholine levels using a cholinesterase inhibitor (ChEI) such as donepezil can produce a modest symptomatic benefit in patients with AD [1]. Cholinergic deficit parallels worsening of cognitive deficits despite treatment with a ChEI [3].

Currently available biomarkers of AD are nonspecific, invasive, and/or expensive. There is a need to develop reliable, noninvasive, safe, and cheap biomarkers for AD that can assess disease progression over time. Such biomarkers will be invaluable in confirming the diagnosis and could act as a quantitative and/or objective marker of potential therapeutic benefit in clinical trials.

Transcranial magnetic stimulation (TMS) is a noninvasive, relatively cheap neurophysiological technique that assesses the excitability of the motor cortex. A specific paradigm—short-latency afferent inhibition (SAI), which couples TMS with a conditioning electric stimulus on the peripheral nerve—has been designed to measure the excitability of cholinergic circuits within human cerebral motor cortex [4], [5]. This results in a reduction in amplitude of motor evoked potentials (MEPs) obtained with TMS. SAI is abolished with administration of intravenous scopolamine, suggesting that it is at least partly mediated by cortical cholinergic activity [5]. In a small study, AD patients had reduced SAI compared to control subjects reflecting their cholinergic dysfunction [6]. In a subgroup of six AD patients in the same study, SAI increased after a single dose of rivastigmine, a ChEI [6]. This study aimed to assess whether SAI can be used as a biomarker of AD pathology and response to treatment with a ChEI.

## 2 Materials and methods

### 2.1 Participants

Nineteen AD patients meeting the consensus criteria for typical mild-to-moderate AD [7] treated with donepezil once daily and 20 healthy subjects (HS) participated in the study. Their demographics and clinical characteristics are summarized in Table 1. This study has been approved by the appropriate local and national research ethics committees, and all participants gave informed consent in accordance with the Declaration of Helsinki.

**Table 1:** Demographic and neurophysiological profiles of the participants

	Control	AD
Gender (M:F)	9:11	9:10
Age (years ± SD)	66.3 ± 7.0	71.0 ± 8.1
Handedness (R:L)	19:1	19:0
MMSE (/30 ± SD)	29.8 ± 0.5	23.4 ± 3.3
MMSE < 24	0	11
Symptom duration (years ± SD)	N/A	4.0 ± 2.0
Range of symptoms (years ± SD)	N/A	2–8
Donepezil taken (night:day)	N/A	12:8
Donepezil dose (10 mg:5 mg)	N/A	18:1∗
Donepezil treatment duration (years ± SD)	N/A	1.7 ± 1.1
Interval between 2 visits (days ± SD)	13.7 ± 1.0	14.4 ± 2.3
Sensory threshold (mA ± SE)		
Morning	2.60 ± 0.96	2.80 ± 1.08
Afternoon	2.55 ± 0.78	2.88 ± 0.91
SEP stimulation intensity (mA ± SE)		
Morning	7.79 ± 2.89	8.36 ± 3.30
Afternoon	7.64 ± 2.45	8.78 ± 2.74
N20 latency (ms ± SE)		
Morning	20.43 ± 1.24	20.26 ± 1.42
Afternoon	20.44 ± 1.43	20.11 ± 1.45
N20-P25 amplitude (μV ± SE)		
Morning	6.19 ± 3.28	6.37 ± 3.53
Afternoon	6.45 ± 3.32	6.54 ± 3.88
Resting motor threshold (%MSO ± SE)		
Morning	47.65 ± 8.17	49.24 ± 12.22
Afternoon	47.60 ± 9.84	49.95 ± 12.70
1 mV motor threshold (%MSO ± SE)		
Morning	64.41 ± 14.44	71.74 ± 17.89
Afternoon	62.49 ± 13.74	72.34 ± 21.09

Abbreviations: AD, Alzheimer's disease; SD, standard deviation; SE, standard error; M, male; F, female; R, right; L, left; MMSE, Folstein Mini–Mental Status Examination; N/A, not applicable; SEP, sensory evoked potential; MSO, maximum stimulator output.

NOTE. Resting motor threshold and 1-mV motor threshold are measured as a percentage of the maximum stimulator output.

∗ One patient on 5 mg did not tolerate titration to 10 mg and was stepped down to 5 mg several months before enrollment in this study.

### 2.2 Experimental procedures

SAI was tested four times over two separate visits in all participants. There were two sessions per visit, 4 hours apart (“morning” and “afternoon” sessions). The AD group was asked to delay their daily dose of donepezil preceding the visit until immediately after the completion of the morning TMS session, for an interval of at least 24 hours between donepezil ingestion and the morning TMS session. This timing was based on the pharmacokinetic properties of donepezil to create a relatively deficient cholinergic state for the morning session, compared to the afternoon session occurring 4 hours later, when the serum level of donepezil is expected to be at its peak [8], [9].

### 2.3 Resting motor threshold and SAI

Resting motor threshold was defined as the lowest TMS intensity applied over the left primary motor cortex able to evoke an MEP of at least 50 μV in 5 of 10 consecutive trials during rest in the right first dorsal interosseous muscle. SAI was performed using the method previously described by Tokimura et al. [4]. Briefly, SAI was obtained by coupling a TMS pulse applied over primary motor cortex, at an intensity able to elicit a MEP of around 1 mV amplitude from the right first dorsal interosseous (1 mV-int), with an electric stimulus of 200 μs duration and a somatosensory stimulation intensity (SST) of 300% of the somatosensory threshold over the right median nerve. The interstimulus interval (ISI) between electric and magnetic pulse was adjusted based on individual latency of the N20 component of somatosensory evoked potentials recorded over the left hemisphere according to current guidelines [10], using the same SST of SAI. Fifteen trials for single, control TMS pulse and for each ISI (N20 +2, +4, +6 and +8 ms) were collected in a randomized order. SAI was then calculated as the ratio between the averages of conditioned and control MEP at each ISI.

### 2.4 Statistical analysis

Age and neurophysiological variables (SST, somatosensory evoked potential N20 latency, somatosensory evoked potential N20-P25 amplitude, resting motor threshold, and 1 mV-int) were compared between AD patients and control subjects by means of two-way mixed analyses of variance (ANOVAs) with “group” and “session” as factors of analysis. A two-way ANOVA with “group” and “session” as factors of analysis was used to compare the test MEP amplitudes. A mixed three-way ANOVA with “group,” “session,” and “ISI” as factors of analysis was performed on SAI ratios to investigate possible SAI changes between HS and AD patients in different sessions and ISI. Several other three-way repeated-measures ANOVAs with “session,” “muscle,” and “ISI” as factors of analysis were performed on SAI ratios in the AD group adding disease severity and time of donepezil intake as covariates. Pearson's correlation coefficient was used to investigate possible correlations between SAI ratios (for each ISI and averaged across ISIs) and clinical variables (disease duration and Mini–Mental Status Examination values) in AD patients. To assess the reproducibility of SAI, Pearson's correlation coefficient was used to investigate correlation between SAI values, averaged across the four ISI, in the four different sessions in HS and AD patients separately. Normality of distribution was assessed with the Shapiro-Wilks' test, whereas homogeneity of variance was assessed by Levene's test. Greenhouse-Geisser correction was used where applicable in case of violation of sphericity. *P* values < .05 were considered significant. Bonferroni correction was used for post hoc comparisons.

## 3 Results

All variables were normally distributed (*P* values of the Shapiro-Wilks' test were always > .05), and Levene's test did not disclose violation of homogeneity of variance. The multiple two-way mixed ANOVAs did not show any main effects or interactions of factors “group” and “session” when comparing SST, N20 latency, N20-P25 amplitude, and resting motor threshold (Table 1). 1 mV-int was larger in AD patients than in HS, although the main effect of the “group” factor of the related ANOVA did not reach statistical significance (F_1,35_ = 3.085, *P* = .088) and there were no factor interactions. The two-way mixed ANOVA with factors “group” and “session” done to compare test MEPs did not disclose significant main effects or interactions. The three-way mixed ANOVA with “group,” “session,” and “ISI” factors on SAI ratio only disclosed a significant main effect of “ISI” (F_3,102_ = 71.530, *P* < .001); no other main effects or factor interactions were observed. Post hoc comparisons showed that shorter ISI led to significantly greater inhibition compared to longer ones. Time of intake and dose of donepezil did not show any effect as covariates. No correlation was found in AD patients between Mini–Mental Status Examination and SAI measured for each ISI and averaged across all ISIs. Pearson's correlation coefficient showed no correlation in the SAI among the four different sessions in AD patients, whereas a significant correlation was found in the control group between the morning and the afternoon sessions.

## 4 Discussion

**Fig. 1:**
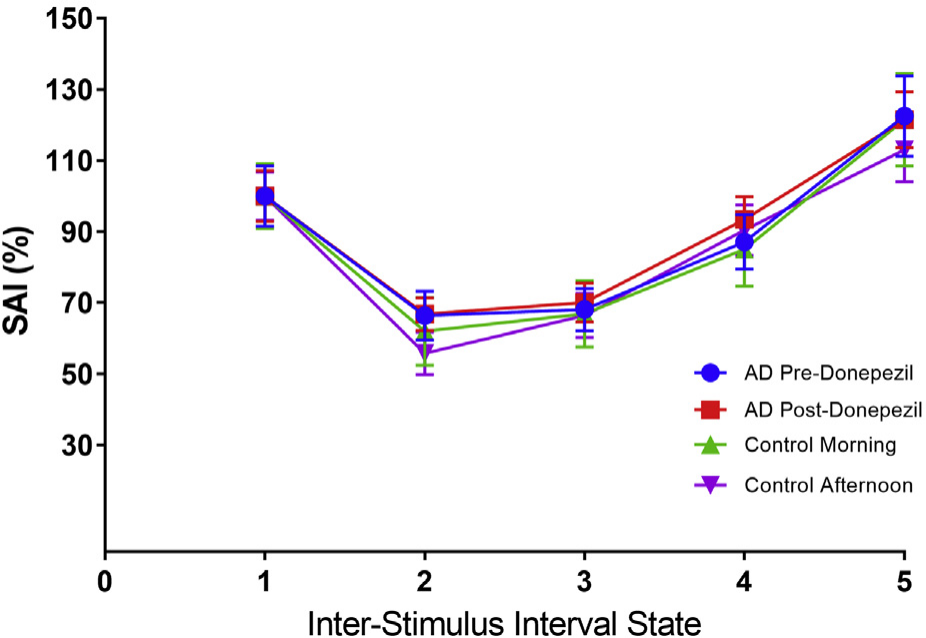
Short-latency afferent inhibition associated with each interstimulus interval state. The averages of the transcranial magnetic stimulation (TMS) evoked motor potentials in mV for each state expressed as a percentage of the control pulse for each experimental group across both days. The error whiskers represent the standard error. Each interstimulus interval (ISI) was derived from each participant's individual N20 via sensory evoked potential. ISI 1 = control state with TMS only; ISI 2 = TMS after the peripheral nerve stimulation at N20 + 2 ms; ISI 3 = TMS after the peripheral nerve stimulation at N20 + 4 ms; ISI 4 = TMS after the peripheral nerve stimulation at N20 + 6 ms; ISI 5 = TMS after the peripheral nerve stimulation at N20 + 8 ms. Abbreviations: AD, Alzheimer's disease; SAI, short-latency afferent inhibition.

In our study, SAI was not able to distinguish the cholinergic deficit state in AD patients compared with HS (Fig. 1). Furthermore, SAI did not detect change in the cholinergic state before and after donepezil administration in AD patients. Interestingly, correlation in SAI among the four sessions was significant in HS, while it was not in AD patients. This indicates a greater variability of cortical cholinergic transmission in the AD group and raises a possibility that there is a diurnal variability in the cholinergic state in HS, which is lost in AD, either as a result of the underlying neurodegenerative process or because of the treatment boosting acetylcholine labels.

Our results are different from a previous report that found significantly reduced SAI in AD patients compared to HS [6]. The major difference in the study subjects is that AD patients studied by Di Lazzaro et al. [6] were naïve to treatment with ChEI, while our patients had been on donepezil long term (average of 20.8 months) before undergoing SAI 24–36 hours after the last dose of ChEI. A modicum of cholinesterase inhibition still present in our subjects may be sufficient to restore SAI to levels indistinguishable to a normal state suggesting that at least pharmacologically, the ceiling effect of ChEI may be attained at lower doses of ChEI. Another possible explanation, previously reported in rats [11], is that chronic donepezil administration induces neuroplastic changes in the brain to adapt to the damages acquired from the neurodegenerative disorders, although it is not known whether such changes occur in humans at this stage.

The results of our study indicate that SAI is not sufficiently sensitive to differentiate between the deficient and normal cholinergic states in AD patients on long-term therapy with ChEI and HS or detect a change in the cholinergic state before and after the administration of the daily dose of donepezil in these AD patients. The results suggest that SAI is unlikely to be a useful biomarker that can reliably detect changes in cholinergic function to distinguish ChEI responders from nonresponders or as an effect of treatment with other symptomatic or disease-modifying treatment for AD above and beyond ChEI. However, the loss of correlation between SAI in the four sessions, which was present among HS, warrants further investigation. If these intriguing results are replicated, then this may indicate the loss of normal diurnal variability of cholinergic function in AD patients, compared to unaffected individuals, potentially providing new insights into pathological biochemical process underlying AD.

**Research in Context**

1. Systematic review: The authors reviewed the literature using traditional sources (e.g., PubMed). There are no reports assessing the response to treatment with donepezil in Alzheimer's disease (AD) with transcranial magnetic stimulation (TMS); the existing literature focuses on the potential role of TMS in AD diagnosis. The relevant articles have been cited appropriately in the text.
2. Interpretation: Our finding suggests that TMS is not sufficiently sensitive to distinguish between control participants and AD patients on chronic donepezil therapy.
3. Future directions: Despite the negative results, our study also suggests two new, intriguing avenues to explore: the existence of a diurnal variation in acetylcholine activity in healthy controls, which is lost in AD; and whether neuroplastic changes, detectable with TMS, occur in AD in response to chronic donepezil administration. This could provide additional insights into biochemistry and pathophysiology of AD.
